# Management of Recurrent Rectourethral Fistula by York Mason Posterior Transrectal Transsphincteric Approach

**DOI:** 10.1155/2015/854365

**Published:** 2015-12-06

**Authors:** Fahri Yetişir, A. Ebru Şarer, H. Zafer Acar, Omer Parlak, Gokhan Osmanoglu, Gulen Karalova

**Affiliations:** ^1^General Surgery Department, Atatürk Research and Training Hospital, Turkey; ^2^Anesthesiology and Reanimation Department, Atatürk Research and Training Hospital, Turkey; ^3^Bozok University General Surgery Department, Turkey; ^4^General Surgery Department, Medical Park Private Hospital, Turkey

## Abstract

Rectourethral fistula (RUF) may develop after ureterovesical and rectal intervention or radiation therapy (RT) rarely, but it is associated with significant morbidity and mortality. The patient will typically present with pneumaturia, faecaluria, and urinary drainage from the rectum. Diagnosis can be easily done with digital rectal examination, cystography, and urethrocystoscopy. Conservative supportive management of RUF does not appear to be successful in most patients, and management with surgical intervention remains the best treatment option. Several surgical techniques have been described including transabdominal, transanal, transperineal, combined abdominoperineal, anterior and posterior transsphincteric, transsacral, laparoscopic, robotic, and endoscopic minimally invasive approaches. There have been very few data about treatment of recurrent RUF. We would like to report the management of recurrent RUF following transurethral resection of prostate and RT for prostate carcinoma in an immunosuppressed, 75-year-old patient by York Mason posterior transrectal transsphincteric approach.

## 1. Introduction

Rectourethral fistula (RUF) is uncommon disorder but is associated with significant morbidity and mortality. RUF may occur after various conditions including urologic malignancy, pelvic trauma, inflammatory bowel disease, rectal surgery, and chronic infection [1]. The most common etiology of RUF is prostate procedures such as radical prostatectomy, transurethral resection of prostate (TURP), radiotherapy (RT), brachytherapy (BT), cryotherapy, or video-laparoscopic radical prostatectomy (VLS-RP) [1, 2]. The patient will typically present with pneumaturia, faecaluria, and urinary drainage from the rectum. Fever and fatigue are also common symptoms [3]. The incidence of RUF after radical prostatectomy is less than 2%, 0.2% for brachytherapy, and 2.9% for combined external beam therapy and brachytherapy boost [1].

The diagnosis of RUF is not difficult; it can be easily palpated in the anterior rectal wall by digital rectal examination (DRE). RUF can also be seen using cystourethroscopy, colonoscopy, or a contrast study of the rectum [3].

Conservative supportive management of RUF does not appear to be successful in most patients, and management with surgical intervention remains the best treatment option [2]. Several surgical techniques have been described including transabdominal, transanal, transperineal, combined abdominoperineal, anterior and posterior transsphincteric, transsacral, laparoscopic, robotic, and endoscopic minimally invasive approaches. There has been no reported data available clearly favoring one approach [1, 2]. Surgical repair of RUFs is challenging and there is no a standardized approach, due to the rarity of RUF and the heterogeneity of fistula etiology and morphology [1]. There have been very few data about treatment of recurrent RUF.

We would like to report the management of recurrent RUF following TURP and RT for prostate carcinoma in an immunosuppressed patient by York Mason posterior transrectal transsphincteric approach.

## 2. Case Presentation

**Figure 1:**
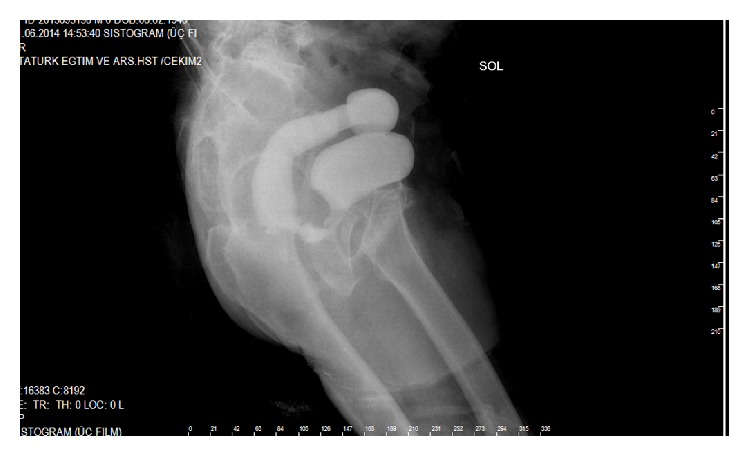
Cystographic presentation of RUF between rectum and urethra is seen.

**Figure 2:**
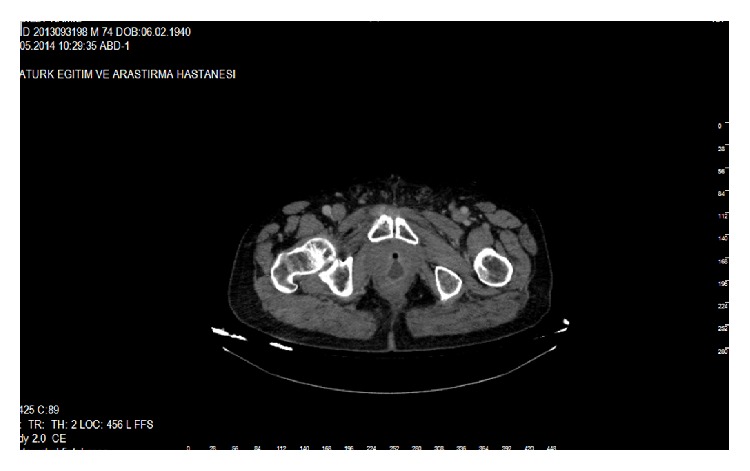
Rectum, urethra, and RUF are seen in tomographic view.

A 75-year-old man was admitted to our department with the complaint of pneumaturia, faecaluria, and urinary drainage from the rectum and fever. In his past history, he was using Prednol 16 mg tablet for arthritis and insulin for diabetes mellitus. TURP had been applied for benign prostate hyperplasia. External beam RT had been applied after pathologic result coming as prostate carcinoma. 45 days after RT, TURP had been repeated due to occlusion. The patient noticed all these complains one week after this operation. RUF was diagnosed by DRE and confirmed by cystourethroscopy. Cystostomy and a loop colostomy had been performed 25 days after the second TURP. After this operation patient's complain continued with a little decrease. Six months after this operation transperineal repair of the RUF with gracilis muscle flap transposition was performed. One month after this RUF repair, he was readmitted to our hospital with his previous complains including pneumaturia, faecaluria, and urinary drainage from the rectum and fever. The recurrent RUF was palpated by DRE. It has been observed that the enteric influent passing to the distal part of colostomy and distal opening of loop colostomy was closed by Vicryl suture. After this operation patient was hospitalized several times for urinary sepsis. Six months after this operation RUF was palpated by DRE and the cystogram demonstrated the persistence of the fistula with approximately 2 cm in diameter (Figure 1). The computed tomography confirmed the presence of a fistulous tract between the urethra and the rectum (Figure 2).

## 3. Surgical Procedure

**Figure 3:**
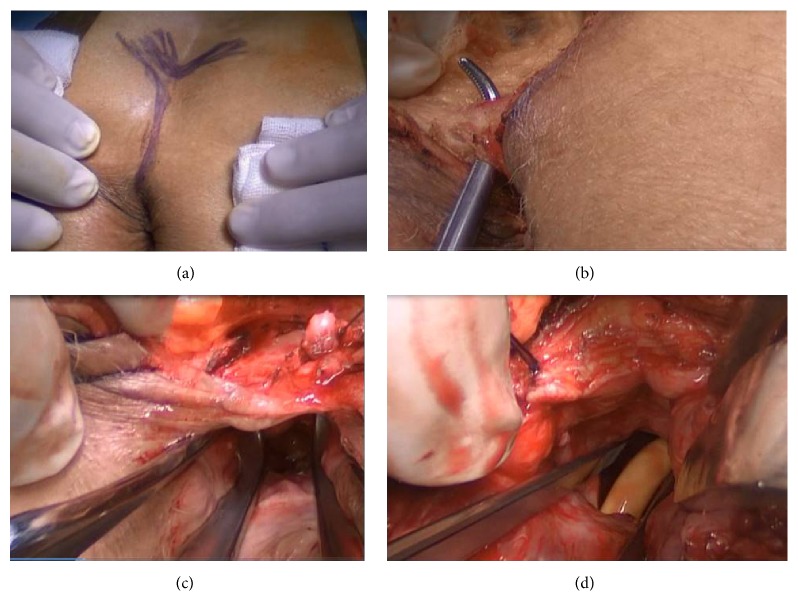
Pictures showing the opening of the posterior rectal wall; it is first part of the York Mason posterior transrectal transsphincteric procedure. (a) Incision line is seen. (b) After opening skin and subcutaneous tissue, gluteus and anal sphincter muscles are seen. (c) Incised anal sphincter muscles and posterior rectal wall are seen. (d) After opening posterior rectal wall, the rectourethral fistula (RUF) and urethral catheter are seen.

**Figure 4:**
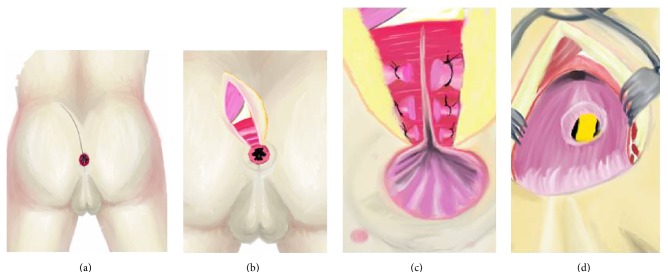
Schematic illustration of the picture in Figure 1 showing opening of the posterior rectal wall; it is first part of the York Mason posterior transrectal transsphincteric procedure. (a) Incision line is seen. (b) After opening skin and subcutaneous tissue, gluteus and anal sphincter muscles are seen. (c) Incised anal sphincter muscles and posterior rectal wall are seen. (d) After opening posterior rectal wall, the rectourethral fistula (RUF) and urethral catheter are seen.

**Figure 5:**
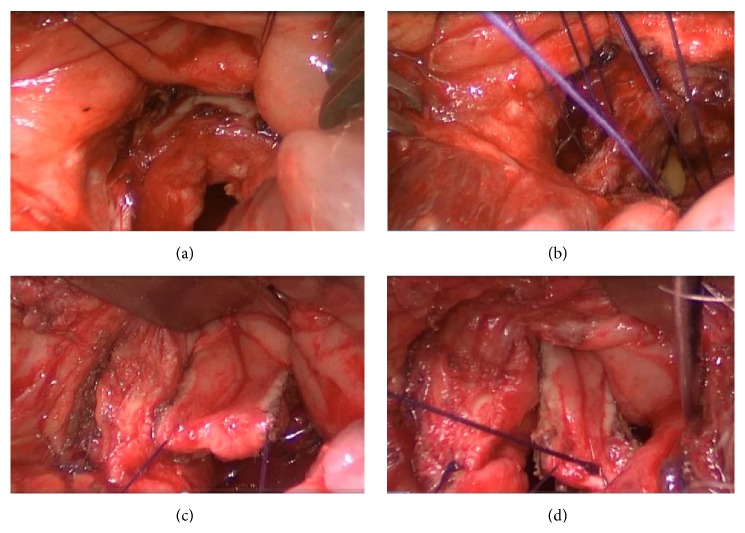
Pictures showing the dissection and closure of RUF. (a) All through the anterior rectal wall and posterior urethral wall were separated by sharp dissection. (b) After separation, first closure of the urethral wall by interrupted suture is seen. (c) After finishing posterior urethral wall closure, prepared rectal advancement flap from proximal side is seen. (d) After preparing rectal advancement flap, its fixation with interrupted sutures one by one is seen.

**Figure 6:**
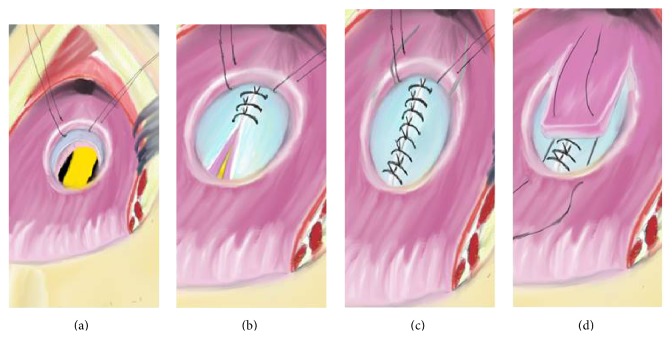
Schematic illustration of the picture in Figure 5 showing dissection and closure of RUF. (a) All through the anterior rectal wall and posterior urethral wall were separated by sharp dissection. (b) After separation, first closure of the urethral wall by interrupted suture is seen. (c) After finishing posterior urethral wall closure, prepared rectal advancement flap from proximal side is seen. (d) After preparing rectal advancement flap, its fixation with interrupted sutures one by one is seen.

**Figure 7:**
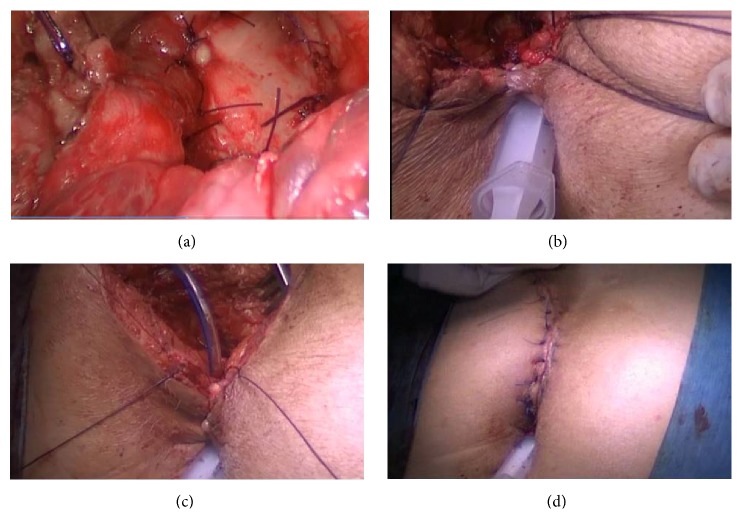
Pictures showing closure of posterior rectal wall and anal sphincter in an anatomical plane. (a) Closed RUF with rectal flap is seen. (b) Closed posterior rectal wall is seen. (c) Separately repaired anal sphincters by the guidance of suture which was placed at the beginning of operation. (d) Closed skin incision of the York Mason operation is seen.

**Figure 8:**
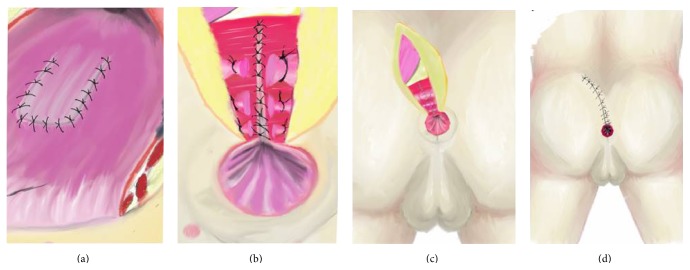
Schematic illustration of the picture in Figure 7 showing closure of posterior rectal wall and anal sphincter in an anatomical plane. (a) Closed RUF with rectal flap is seen. (b) Closed posterior rectal wall is seen. (c) Separately repaired anal sphincters by the guidance of suture which was placed at the beginning of operation. (d) Closed skin incision of the York Mason operation is seen.

Preoperative intravenous antibiotic prophylaxis with levofloxacin 500 mg plus teicoplanin 400 mg was administered. The procedure began with lithotomy position. The scar of previous transperineal gracilis transposition operation was seen. RUF was visualized again and an 18-F Foley transurethral catheter was placed by the help of cystourethroscopy. Then position of the patient was changed to the prone in the Jackknife position and the buttocks were separated with adhesive tape. The incision was made from the level of the anal margin to the approximately 30°C right side of the tip of the coccyx. Several bilateral sutures were placed in the anal sphincter to guarantee adequate reconstruction of the anus. At this point, both the internal and the external sphincteric and puborectal muscles were carefully identified with suturing and incised. Then, the posterior wall of the rectum was incised to expose the anterior rectal wall (Figures 3 and 4). With the York Mason approach, the orifice of the fistulous tract was easily visualized; the urethral and the anterior rectal walls were separated from each other by sharp dissection. A plane of dissection was created between the rectal and bladder walls to ensure tension-free closure. Vicryl 2-0 and PDS 3-0 sutures were used to close the urethra; then anterior rectal wall was closed with advancement flap from proximal mucosa (Figures 5 and 6). After that posterior rectal wall was closed with 2-0 Vicryl suture and the anal sphincter's structures were repaired one by one by the guidance of the sutures placed at the beginning of the surgery. An aspiration drain was left in the subcutaneous layer. The skin was sutured with polypropylene 3-0 suture (Figures 7 and 8).

## 4. Follow-Up

**Figure 9:**
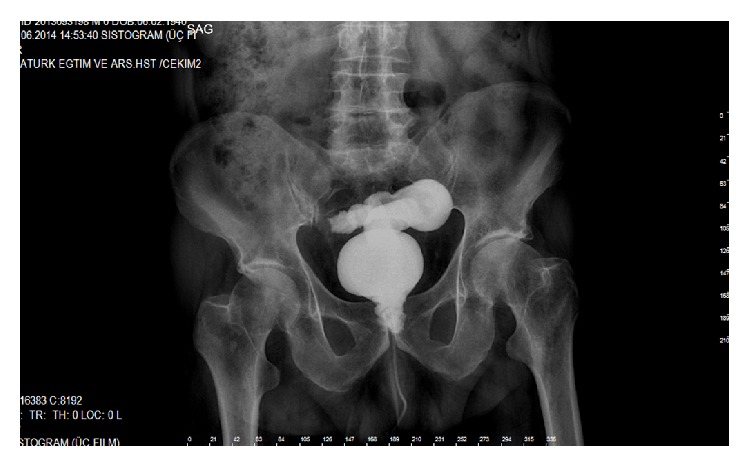
Leakage of radiopaque material filling the rectum by passing through rectourethral fistula was seen preoperatively in cystography.

**Figure 10:**
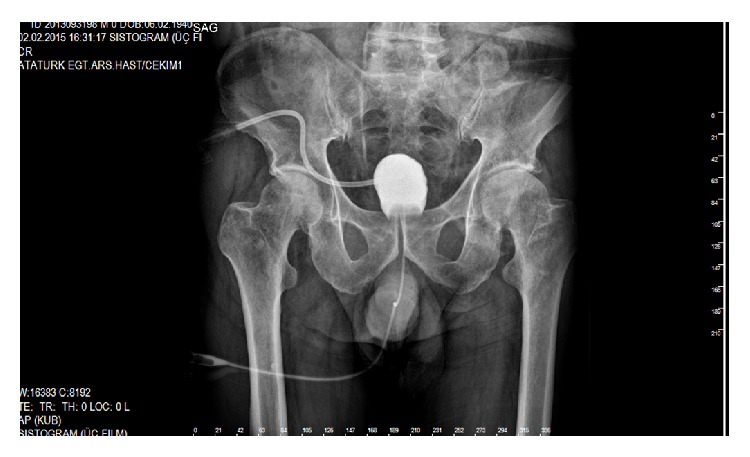
It was seen that there was no fistula any more between the urethra and rectum in postoperative cystography 3 months after operation.

Oral intake was resumed after recovery from anesthesia. Drain is usually removed by 3 days after surgery. At postoperative period wound infection was developed and healed with local negative pressure therapy (NPT). On the 10th postoperative day he was discharged. A transurethral and cystostomy catheter was maintained for 3 weeks. The patient was followed up by clinical examination and cystography 9 months after the operation; there was no recurrence and incontinence sign. Leakage of radiopaque material filling the rectum by passing through rectourethral fistula was seen preoperatively in cystography (Figure 9), but not postoperatively (Figure 10). Ostomy reversal was planned to be closed 3 months later because of the systemic disease of the patient.

## 5. Discussion

Management of RUF is challenging for the surgeon because spontaneous closure is very rare and the recurrence rate is high. Conservative management with urinary catheter drainage, bowel rest, and intravenous alimentation is usually ineffective [2]. At the beginning, a minimally invasive approach and supportive medical therapy should be attempted. In fact, some favorable results have been reported with application of fibrin glue, endoscopic suturing, or fulguration of the fistulous tract for selected patients with RUF [4].

Keller et al. determined the surgical approach based on five factors: severity of presenting symptoms, fistula size (>1 cm), extent of tissue damage from radiation or cryotherapy, status of the urethra, and presence of active pelvic sepsis at presentation [1]. In some cases RUF may persist or recur after surgical repair. In addition to Keller's five factors, immunosuppressive therapy and applied surgical technique influence the recurrence rate of RUF. A diverting colostomy or ileostomy should be applied as a first step for management of this kind of RUF and tissue interposition to separate the rectal and the urethral sutures, such as the dartos flap and the gracilis muscle transposition that can fill the dead space created by rectal and bladder separation with transabdominal, abdominoperineal, and transperineal approaches [5, 6]. Zmora et al. reported the gracilis muscle transposition for RUF in 11 patients after surgery or pelvic RT for prostate cancer: two (18.2%) patients required further surgical therapy [7]. In our case, prior diverting loop colostomy, cystostomy, and transperineal approach with gracilis muscle transposition was performed in first operation as in the literature advice, due to the fact that his RUF was more than 2 cm in diameter, occurring after RT and surgery, and he was using Prednol for arthritis. Despite all these invasive approaches recurrence of RUF developed one month after operation. Six months later recurred RUF was repaired with York Mason posterior transrectal transsphincteric procedure.

The English surgeon Aubrey York Mason started using a transsphincteric exposure for rectal operations around 1960, and the principles of the York Mason technique were published in 1970 [8]. York Mason approach has become one of the most preferred techniques for management of RUF because this approach is rapid and allows superb exposure of tissue planes with a scarless dissection for a successful surgical intervention, and reported recurrence rate of RUF with this approach is not high [2]. Fecal incontinence is one of the challenging and feared complications after York Mason procedure that involves sectioning the anal sphincter, but fecal incontinence has been reported in the literature in less than 1% of cases [9]. In our case, the recurrence and fecal incontinence have not been developed at the 9th month of follow-up.

## 6. Conclusion

Treatment of RUF after RT and surgery for prostate carcinoma in an elderly immunosuppressed patient is not so easy.

One of the important points for treatment of recurrent RUF is prior diverting loop colostomy. The second one is changing one surgical approach to another; for this case, the first surgical approach for RUF is transperineal approach with gracilis muscle transposition; after RUF recurrence the York Mason posterior transrectal transsphincteric procedure is used as a second surgical approach for treatment of recurrent RUF.
